# Glial Cell Line-Derived Neurotrophic Factor Family Ligands, Players at the Interface of Neuroinflammation and Neuroprotection: Focus Onto the Glia

**DOI:** 10.3389/fncel.2021.679034

**Published:** 2021-06-17

**Authors:** Anastasiia Kotliarova, Yulia A. Sidorova

**Affiliations:** Laboratory of Molecular Neuroscience, Institute of Biotechnology, HiLIFE, University of Helsinki, Helsinki, Finland

**Keywords:** neurotrophic factors, glial cell line-derived neurotrophic factor (GDNF), GDNF family ligands, receptor tyrosine kinase RET, RET agonist, neuropathic pain, neuroinflammation, glia

## Abstract

Well-known effects of neurotrophic factors are related to supporting the survival and functioning of various neuronal populations in the body. However, these proteins seem to also play less well-documented roles in glial cells, thus, influencing neuroinflammation. This article summarizes available data on the effects of glial cell line derived neurotrophic factor (GDNF) family ligands (GFLs), proteins providing trophic support to dopaminergic, sensory, motor and many other neuronal populations, in non-neuronal cells contributing to the development and maintenance of neuropathic pain. The paper also contains our own limited data describing the effects of small molecules targeting GFL receptors on the expression of the satellite glial marker IBA1 in dorsal root ganglia of rats with surgery- and diabetes-induced neuropathy. In our experiments activation of GFLs receptors with either GFLs or small molecule agonists downregulated the expression of IBA1 in this tissue of experimental animals. While it can be a secondary effect due to a supportive role of GFLs in neuronal cells, growing body of evidence indicates that GFL receptors are expressed in glial and peripheral immune system cells. Thus, targeting GFL receptors with either proteins or small molecules may directly suppress the activation of glial and immune system cells and, therefore, reduce neuroinflammation. As neuroinflammation is considered to be an important contributor to the process of neurodegeneration these data further support research efforts to modulate the activity of GFL receptors in order to develop disease-modifying treatments for neurodegenerative disorders and neuropathic pain that target both neuronal and glial cells.

## Introduction

Neurotrophic factors (NTFs) support the development, survival, and functioning of different neuronal populations via activation of intracellular signaling cascades important for well-being of the cells. Currently four families of proteins are being referred to as NTFs: neurotrophins, neurokines, glial cell line-derived neurotrophic factor (GDNF) family ligands (GFLs), and cerebral dopamine neurotrophic factor (CDNF)/mesencephalic astrocyte-derived neurotrophic factor (MANF). Members of the first three families of NTFs signal via transmembrane kinase or kinase-associated receptors (Sidorova and Saarma, 2016; Sidorova et al., 2019). The mechanism of CDNF/MANF signaling is less well-studied, they are endoplasmic reticulum associated proteins and may interact with lipids. Some studies suggest that the entry of CDNF and MANF into the cell as well as their biological effects can be mediated by lipids (Bai et al., 2018). Recent study also showed that transmembrane protein neuroplastin, belonging to immunoglobulin superfamily, may serve as a receptor for MANF and mediate anti-inflammatory effects of this NTF (Yagi et al., 2020).

Although the effects of NTFs were originally evaluated in neuronal cells, growing body of evidence indicates that they may also influence glial cells and modulate neuroinflammation. In the current review we will focus mainly on the role of GFLs and their receptors in sensory neurons and glial cells in neuropathic pain, but also mention some other NTFs.

Neurotrophins, neurokines and GFLs have been extensively studied in sensory system and in neuropathic pain (Khan and Smith, 2015; Eapen et al., 2019; Mahato and Sidorova, 2020a).

According to a definition given by International Association of the Study of Pain (IASP) neuropathic pain is a “pain caused by a lesion or disease of the somatosensory nervous system” (Murnion, 2018). Current view on pathogenesis of neuropathic pain implies the interplay of several cell types including at least sensory neurons, glial cells, peripheral immune cells and target-derived cells. Nerve lesion activates glial and immune cells which start to secrete various proinflamatory cytokines, growth factors and other inflammatory mediators which sensitize peripheral and central neurons (Ji X.-T. et al., 2013).

The role of neurotrophins and neurokines in neuroinflammation is well-established (Davis et al., 2018; Lima Giacobbo et al., 2019; Parker et al., 2019; Hu et al., 2020; Yin et al., 2020). These proteins are also mainly pronociceptive although some of them can have antinociceptive effects as well (Khan and Smith, 2015; Hu et al., 2020). A good summary of neurotrophins' actions in the sensory system can be found in the review of Khan and Smith (2015). According to the available information CDNF and MANF suppress neuroinflammation (Nadella et al., 2014; Zhang et al., 2019) and may promote nerve regeneration (Lindholm et al., 2007; Zhao et al., 2016) and repair lesioned tissues (Neves et al., 2016; Tseng et al., 2018; Eesmaa et al., 2021), however, their effects in sensory system at the moment are poorly documented. Anti-inflammatory effects of MANF in human beta cells are mediated by downregulation of cytokine expression (IL-1b and TNF-a) (Hakonen et al., 2018). In a model of traumatic brain injury MANF also suppressed expression of proinflammatory cytokines and reduced the disruption of blood brain barrier. On molecular level MANF downregulated NF-κB signal transduction pathway (Li et al., 2018). Since these mechanisms are rather universal for inflammatory responses in the body further studies on the effects of MANF and CDNF in somatosensory system in neuropathic pain models are warranted.

Scientific literature on biological effects of GFLs in neuropathic pain contains variable data (Sah et al., 2005; Ossipov, 2011; Merighi, 2016; Ferrini et al., 2020, Cortés et al., 2017). While in rats in injury-based models of neuropathic pain these proteins seem to exert mainly analgesic effects, in inflammatory pain models and in mice they often induce hyperalgesia and/or allodynia. Administration schedule and duration of the treatment with GFL also seem to influence the outcome (Mahato and Sidorova, 2020a). In recent clinical trials in patients with neuropathic pain one of GFLs, artemin (ARTN, also known as enovin and neublastin), was found to be safe and relatively well-tolerated (Rolan et al., 2015; Okkerse et al., 2016). Moreover, it exhibited analgesic effects in a cohort of patients with neuropathic pain resistant to the treatment with standard analgesics (Backonja et al., 2017).

While the main mechanism of GFLs effects in neuropathic pain is considered to appear as a result of trophic support and neurorestoration in somatosensory system, these proteins may also modulate neuroinflammation. This effect can be secondary and appear as a result of restoration of injured sensory neurons, which in turn reduces neuroinflammation, but can also be mediated by a direct action of GFLs in glial cells. In the present review we at first will list the cell types playing a role in neuropathic pain and depict their interactions. Afterwards we will discuss the effects of GFLs in neuronal and non-neuronal cells in somatosensory system. We will also describe the expression of GFLs receptors in various cell types in somatosensory system and present own data showing downregulation of satellite glial marker in dorsal root ganglia (DRGs) upon treatment with GFLs or GFL receptors agonists in animals with experimental neuropathies.

## Cell Types Playing a Role in Neuropathic Pain

In early studies, neuropathic pain was associated exclusively with neuronal dysfunction (Zhuo, 2007). Therefore, the first-line treatments for neuropathic pain are mainly aimed at reducing the excitability of neurons by modulating the activity of ion channels (gabapentinoids) or enhancing endogenous inhibitory mechanisms (tricyclic antidepressants and serotonin-noradrenaline reuptake inhibitors) (Attal and Bouhassira, 2015; Finnerup et al., 2015).

In general in acute and neuropathic pain signal from nociceptors does not immediately damage neurons, the nociceptors thresholds are lower than the stimulus intensity needed to damaged tissues, and, once activated, they release a cocktail of fast-acting (glutamate) and slow-acting neurotransmitters (peptides, trophic factors) from their central terminals (Merighi, 2016).

In the concept of neuronal dysfunction NMDA receptors, which play a critical role in synaptic plasticity in pain transmission pathways may represent a key component. However, in practice, targeting NMDA receptors turned out to be complicated. Non-selective NMDA antagonists produce a number of side effects that limit their clinical use and contribute to treatment failure (Aiyer et al., 2018). NMDA receptor antagonists selective for the N-methyl D-aspartate receptor subtype 2B (NR2B) (Kim et al., 2012) produce less adverse events (Wu and Zhuo, 2009; Zhang et al., 2009), but their efficacy remains to be improved (Boyce et al., 1999; Dahanl et al., 2011; Swartjes et al., 2011).

**Figure 1:**
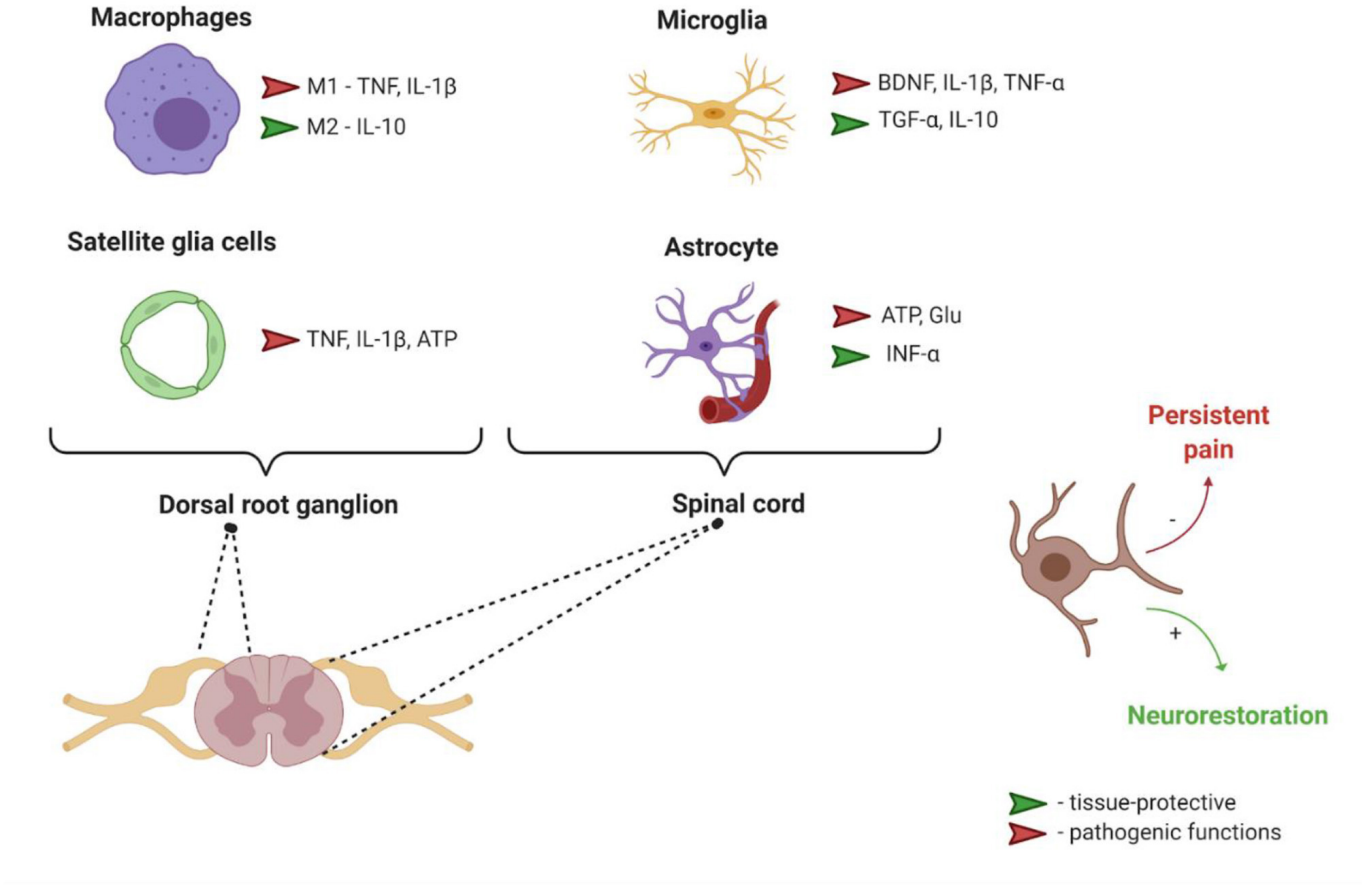
Interaction between nociceptors and different non-neuronal cells. ATP, Adenosine triphosphate; BDNF, Brain derived neurotrophic factor; Glu, glutamate; IL-1β, Interleukin 1 β; IL-10, Interleukin 10; M1, pro-inflammatory phenotype; M2, anti-inflammatory phenotype; TGF-α, Transforming growth factor α; TNF-α, Tumor necrosis factor α.

Although the pathophysiology of neuropathic pain is still controversial, an important step forward in understanding its mechanisms was the realization that neurons are not the only cell type involved in the etiology of this condition. Macrophages, satellite glial cells, microglia and astrocytes, all these non-neuronal cells produce both pro-nociceptive and anti-nociceptive mediators (see Figure 1), which can bind their respective receptors in the nociceptor neurons and modulate their sensitivity and excitability, thus mediating pain transmission in the PNS and CNS.

Macrophages have different phenotypes related to their functional status, including pro-inflammatory M1-like and anti-inflammatory M2-like phenotypes (Martinez and Gordon, 2014). These cells are involved in modulation of pain through the release of pro-inflammatory (such as TNF and IL-1β) or anti-inflammatory mediators (such as IL-10) (Ji et al., 2016).

Satellite glial cells (SGCs) are the main type of homeostatic glial cells in PNS ganglia (sensory, parasympathetic and sympathetic). SGCs are functionally similar to astrocytes (Jasmin et al., 2010). In animal model of intervertebral foraminal stenosis and low-back pain, chronic compression of the DRGs led to an enhancement of glial fibrillary acidic protein expression in SGCs (in the absence of hypertrophy and proliferation usually occurring after sciatic nerve axotomy), an increase in SGCs coupling via gap junctions and a decrease in inwardly rectifying potassium currents. These events were accompanied by increased excitability of DRG neurons. Based on collected data the authors suggested that altered potassium balance in SGCs may lead to the neuronal hyperexcitability (Zhang et al., 2009). SGCs similarly to astrocytes in CNS scavenge extracellular glutamate, however, the increase in the number of gap junctions leads to glutamate recycling thus contributing to the development and/or maintenance of neuropathic pain (Ohara et al., 2008; Jasmin et al., 2010).

Microglial cells are among the key players in the development of neuropathic pain (Block et al., 2007; Suter et al., 2007; Beggs et al., 2012).

Microglia in response to any damage or other abnormal situation in the nervous system initiates an inflammatory response. Due to the presence of immune receptors such as toll-like receptors (TLRs), nucleotide-binding oligomerization domains (NODs), NOD-like receptors, and many scavenger receptors microglial cells (as well as some other cells in the nervous system, e.g., astrocytes) are able to recognize pathological stimuli (Ransohoff and Brown, 2012). In response to these stimuli, microglia releases inflammatory mediators such as tumor necrosis factor (TNF)-α, interleukin (IL)-1, IL-6 and IL-12, interferon-γ (IFNγ) and generates reactive oxygen and nitrogen species (Block et al., 2007; Colton and Wilcock, 2010; Guzman-Martinez et al., 2019), which produce a long-lasting sensory neuron hypersensitivity.

After peripheral nerve injury, molecules released from the spinal cord neurons contribute to spinal microglial activation, causing proliferation and changes in morphology. The key molecule that modulates microglial activity is ATP, an endogenous ligand of the P2 purinergic receptor family. Microglia expresses several subtypes of P2 receptor, and of these, the P2X4 has become the main cross-signaling junction point between microglia and neurons: activation of this receptor stimulates the release of trophic factors and other signaling mediators which in turn trigger neuronal processes such as pain signaling disinhibition (Trang et al., 2011; Ji et al., 2016; Tsuda and Inoue, 2016) and/or neurorestoration.

Importance of microglia in the development of neuropathic pain is also highlighted in the research article of Tsuda et al., where the authors showed that näive spinal microglia of experimental animals expresses a receptor for the cytokine IFN-γ (IFN-γR) in a cell-type-specific manner. Stimulation of this receptor converts resting microglia into activated form and produces a long-lasting hypersensitivity in response to innocuous stimuli (tactile allodynia, a hallmark symptom of neuropathic pain). Conversely, ablating IFN-γR severely impairs nerve injury-evoked microglia activation and tactile allodynia without affecting microglia in the contralateral dorsal horn. At the same time, basal pain sensitivity remains unaffected. The authors also demonstrated an up-regulation of Lyn tyrosine kinase and purinergic P2X4 receptor in IFN-γ-stimulated spinal microglia which are crucial events for neuropathic pain development (Tsuda et al., 2009).

Important role in activation of glial cells in neuropathic pain belongs to BDNF (Ferrini and De Koninck, 2013). Spinal microglia in response to peripheral nerve injury upregulates expression of P2X4 receptors. Activation of P2X4 receptors initiates a signaling cascade which leads to BDNF release which causes aberrant nociceptive output that underlies pain hypersensitivity characterized by hyperalgesia, allodynia, and spontaneous pain (Trang et al., 2011). BDNF produced by microglia forms a positive feedback loop and acts as an autocrine microglia stimulator (Zhang et al., 2014). Also, in experimental morphine-induced hyperalgesia model morphine caused upregulation of P2X4Rs in the spinal cord microglia, driving synthesis and release of BDNF (Ferrini et al., 2013).

In addition, BDNF activates astrocytes which may sensitize the nociceptive pathway through the release of pro-inflammatory factors, such as cytokines, prostaglandins, or neurotrophins, which in turn contributes to neuropathic pain development (Zhang et al., 2011).

Microglia also interacts with astrocytes after sensory nerve injury. Wang et al. (2017) showed that tetanic stimulation of sciatic nerve induces sciatic nerve injury and long-lasting hypersensitivity in rats. In this model IL-18 was released from activated microglia and together with chemokine CX3CL1 activated NF-kB pathway in astrocytes via IL18R and CX3C1R, respectively which resulted in upregulation of the expression of pro-inflammatory cytokines (Wang et al., 2017). In addition astrocyte-derived IL-1β and IL-23 are thought to promote allodynia/behavioral sensitization to nociceptive stimulation, by modulating NMDA receptor activities in post-synaptic neurons (Bian et al., 2014).

Astrocytes perform numerous critical functions in the nervous system including, among many others, neurotransmitter recycling, formation of the blood-brain barrier, regulation of extracellular ion concentrations, and modulation of synaptic transmission.

After nerve injury, astrocytes lose their ability to maintain the homeostatic concentrations of extracellular potassium ions and glutamate, which leads to neuronal hyperexcitability (Ji R.-R. et al., 2013). Astrocytes can also signal directly to neurons through physically coupled networks mediated by gap junctions which facilitate intercellular signal transmission. Gap junction communication is mediated by connexin-43 (Cx43), the predominant connexin expressed in astrocytes. Nerve injury induces persistent up-regulation of Cx43 in astrocytes and switches the function of Cx43 from gap junction communication to paracrine modulation (Chen et al., 2014). This paracrine regulation leads to the increased release of glutamate, ATP, and chemokines by astrocytes. The astrocyte-derived chemokines act as neuromodulators and can potentiate excitatory synaptic transmission in the spinal cord pain circuitry.

A single human astrocyte may form contacts with more than 1 million synapses (Oberheim et al., 2009). The complexity of the connections points to an important role of astrocytes in nervous system that bears further investigation. As compared with microglial activation, astrocyte activation in chronic pain conditions is more persistent and usually occurs later than microglial activation, indicating their contribution to the chronification of pain (Ji R.-R. et al., 2013).

Liu et al. (2020) in a recent review described interactions of microglia and astrocytes in the neurovascular unit using the concept of phenotypic switch between pro-inflammatory (M1 and A1, respectively) and anti-inflammatory (M2 and A2, respectively) phenotypes.

The most-discussed current classification of microglia/macrophages is based on the M1/M2 paradigm, which is related to their pro- and anti-inflammatory properties (Martinez and Gordon, 2014). The M1/M2 paradigm is often criticized as too simplistic (Ransohoff, 2016). However, this paradigm conveniently reflects the most phenotypically distant (polar) differentiation states of macrophages, and the terminology has caught on and is extensively used in research literature (Ransohoff, 2016; Poltavets et al., 2020).

The main idea is that microglia appears to be more sensitive to pathogens; these cells activate and secrete “molecular signals” such as interleukin-1 alpha (IL-1α), TNF-α, and the complement component subunit 1q (C1q) to trigger reactive astrocytes (Liu et al., 2020). Astrocytes in turn release NTFs to support neuronal survival. For instance, Chen et al. (2015) showed that astrogliosis is neuroprotective as a result of NTFs release from astrocytes. Neutralization of supernatant GDNF released from astroglia significantly reduced neuroprotective effect of astroglia in a model of inflammation caused by bacterial inflammogen lipopolysacharide (LPS). Importantly, LPS failed to induce the synthesis and release of TNF-α and iNOS/NO from highly-enriched astroglial culture (Chen et al., 2015). This may suggest that astroglia itself may not have the ability to directly recognize the innate immune stimuli such as LPS. Instead astrocytes rely on microglia which recognize inflammogen and trigger the release of NTFs from astrocytes (BDNF, CNTF, NGF, MANF) in order to counterbalance collateral damage caused by activated microglia and neuroinflammation. This process aims to support neuronal survival (Liddelow et al., 2017; Jha et al., 2019; Pöyhönen et al., 2019).

Although the vast majority of researchers agree on the important role of microglia in the development and maintenance of neuropathic pain in some studies the importance of astrocytes in this process is stressed. For instance, it was shown that in vincristine-evoked chemotherapy-induced neuropathic pain in rats with obvious mechanical allodynia spinal astrocytes rather than microglia were dramatically activated. These data suggest that astrocytes can play a key role in pathophysiology of chemotherapy-induced neuropathic pain and Astrocyte-Cytokine-NMDAR-neuron pathway may be one of the mechanisms underlying chemotherapy-induced neuropathic pain (Ji R.-R. et al., 2013).

To summarize this section, multiple cell types play a role in the development and maintenance of neuropathic pain (Figure 1). It is nearly impossible to isolate neuronal or non-neuronal components playing the leading role in this process. Perhaps a key strategy in neuropathic pain management should be focused on the regulation of interactions between glial cells, including astrocytes and microglia, and neurons (Zhuo et al., 2011). Thus, understanding of communications between various cell types playing roles in neuropathic pain is very important for the development of efficient analgesics (Ji R.-R. et al., 2013; Ji et al., 2016). NTFs or their receptors which, as described in the following sections, are able to influence both neuronal and non-neuronal cells, may represent valuable targets for innovative drugs for neuropathic pain management. GFLs and their receptors (see below) are especially interesting in the context of neuropathic pain as they are, in contrast to other NTFs, were shown to restore all types of sensory neurons and to target their axons into topographically correct regions of spinal cord in a model of dorsal root crush (Harvey et al., 2010). Extensively studied approach focused on the disruption of NGF signaling using monoclonal antibodies or neurotrophin inhibitors is less attractive in this sense as it is not disease-modifying.

## Glial Cell Line-Derived Neurotrophic Factor Family Ligands, Their Receptors and Signaling

GFLs include 4 closely related proteins: GDNF, neurturin (NRTN), artemin (ARTN), and persephin (PSPN) and a distant recently discovered member, a protein called Growth/differentiation factor 15 (GDF15). The main receptor transmitting GFL signals inside neurons is a receptor tyrosine kinase Rearranged in transfection (RET) (Trupp et al., 1996; Mahato and Sidorova, 2020b). GFLs do not bind RET directly, instead they interact with a co-receptor GDNF family receptor alpha (GFRα) or GDNF family receptor α-like (GFRAL). GDNF has highest affinity to GFRα1, but can also signal via GFRα2, NRTN's preferred receptor is GFRα2, ARTN's—GFRα3, PSPN's—GFRα4. In addition, NRTN, ARNT, and PSPN can all exert their biological effects via GFRα1. GDF15 binds to GFRAL and does not seem to have affinity to GFRα co-receptors (Emmerson et al., 2017; Mullican et al., 2017; Yang et al., 2017). Formation of a dimeric ligand-co-receptor complex (stoichiometry 1:2) leads to the recruitment of two monomers of RET into the compex, autophosphorylation of tyrosine residues in RET kinase domain and subsequent activation of multiple signaling cascades and enzymatic pathways important for cellular survival and functional activity (Airaksinen and Saarma, 2002), such as JAK/Stat, PI3K/Akt, MAPK, JNK, RAC1, VAV2, PLCγ (Mulligan, 2014; Zhu et al., 2015).

In addition to RET, GFLs in complex with GFRα co-receptors signal via neural cell adhesion molecule (NCAM) activating Fyn and FAK protein kinases. This mechanism was shown to mediate migration of Schwann cells and neurite outgrowth in hippocampal and cortical neurons (Paratcha et al., 2003). Also GFLs can activate certain events in the cells via surface Heparan Sulfate Proteoglycan, Syndecan−3 in the absence of co-receptors. GFL interaction with Syndecan-3 stimulates Src-dependent signaling and is important for migration of cortical GABA-ergic neurons (Bespalov et al., 2011).

## Effects of Glial Cell Line-Derived Neurotrophic Factor Family Ligands in Primary Sensory Neurons

GDNF and ARTN have well-established roles in somatosensory system. Modulation of their expression levels in the tissues of experimental animals alters the number of sensory neurons in DRGs. In GDNF knockout mice the number of sensory neurons in DRGs is reduced by ~30% and in mice overexpressing GDNF—increased by about 30%. In mice overexpressing ARTN the number of primary sensory neurons is increased by 20% (reviewed in Ernsberger, 2008). Ablation of GDF15 gene in mice also led to the 20% decrease in the number of primary sensory neurons in DRGs (Strelau et al., 2009). However, NRTN overexpression in the skin was not accompanied by the changes in overall number of sensory neurons in DRGs (Wang et al., 2013).

DRG neurons of mammalians express RET and GFRα co-receptors. Expression of RET and GFRα coreceptors in primary sensory neurons highly, but not completely overlap. The number of DRG neurons expressing GFL receptors depends on developmental stage and increases after birth. Approximately 60% of adult rodent DRG neurons in normal conditions are RET positive, upto 50% are GFRα1-positive, up to 33% are GFRα2–positive and upto 40% are GFRα3–positive (Ernsberger, 2008; Mahato and Sidorova, 2020a). In human DRGs higher number of neurons express RET (80%) and GFRα2 (52%) (Josephson et al., 2001). Expression of GFL receptor complex components in primary sensory neurons can change after lesion. In rodents the number of RET-positive and GFRα3-positive cells in DRGs increases, but the number of GFRα2-positive cells decreases. The neurons which in normal conditions express GFRα2 upon injury are converted into GFRα3-positive cells (Wang et al., 2011; Mahato and Sidorova, 2020a).

The data regarding GFLs expression in DRG neurons are somewhat controversial. According to the recent review of Merighi (2018) small portion of DRG neurons (<10%) expresses GDNF protein as shown by immunological methods. At the same time transcriptomics studies failed to detect GDNF mRNA in DRGs. We also failed to reliably detect mRNA of GFLs in uninjured rodent DRGs by RT-qPCR method. This discrepancy can be explained by either contribution of non-transcriptional mechanisms in the GFL protein synthesis or unspecific binding of GDNF antibodies. Indeed, in our hands many commercially available GDNF antibodies produced non-specific staining in the tissues of experimental animals. Interestingly, in DRGs GDNF-immunoreactive neurons were also CGRP-immunoreactive, while RET and GFRα co-receptors expressing neurons are mainly IB4-positive (Merighi, 2018).

In the spinal cord GFL receptors are expressed in lamina IId which receives and integrates input mainly from C-fibers (Merighi, 2018). Anatomic localization of GFL receptors in the spinal cord supports the important role of GFLs in pain signal transmission.

The GDNF co-receptor (GFRα1) can be released from the surface of neurons and glia in its soluble form. In this way GFLs can interact with corresponding GFRαs in solution and then with RET on cell surface thus activating cells expressing only RET (Airaksinen and Saarma, 2002; Jmaeff et al., 2020b).

The effects of GFLs in primary sensory neurons in the context of neuropathic pain are complex. From one hand they support the survival and repair sensory neurons, eliminating lesion and consequently the cause of neuropathic pain. Therefore, these proteins and their receptors serve as attractive therapeutic targets for disease-modifying treatments of NP. However, GFLs also stimulate the expression of various proteins increasing excitability of neuronal cells, such as ion channel subunits and thus potentiate signal appearing as a result of nociceptive stimulation. We recently reviewed effects of GFLs in different chronic pain models in detail. In general, in neuropathic pain GFLs given intermittently are mainly analgesic, however they may be pronociceptive in inflammatory pain states and in healthy animals (Mahato and Sidorova, 2020a).

Interestingly, certain pronociceptive effects of GFLs can be mediated by sensory neurons expressing only GFL co-receptors in the absence of RET. In particular, in DRGs cold stimulation activates TRPM8-positive neurons expressing GFRα3, but lacking RET (Lippoldt et al., 2013, 2016). This means that in certain subpopulations of primary sensory neurons GFLs signal via other known (e.g., NCAM) or yet to be identified receptor(s). GFRα co-receptors are also expressed in non-neuronal cells, which can influence nociceptive response to stimulation. This suggests that some of pronociceptive effects of GFLs can be mediated via RET independent mechanisms and highlights the possibilities to improve tolerability of GFL-based therapeutics, by development of variants selectively targeting RET.

## Effects of Glial Cell Line-Derived Neurotrophic Factor Family Ligands in Glial Cells

Due to the well-established neurotrophic activity of GFLs they have been mainly studied for the ability to protect neurons from neurotoxic or mechanical lesion (Gash et al., 1996; Boucher et al., 2000; Airaksinen and Saarma, 2002; Gardell et al., 2003; Sidorova et al., 2017; Mahato and Sidorova, 2020a). However, several studies demonstrated the ability of these proteins to affect non-neuronal cells involved in inflammatory process which is developing upon the injury occurring in somatosensory system. GFLs receptors GFRα1 and RET are expressed in glial cells and can be upregulated under pathological conditions. GDNF itself is widespreadly expressed in both neuronal and non-neuronal cells in the developing human fetal brain starting from 7 weeks of gestation (Koo and Choi, 2001).

Primary rat glial cells cultures mainly composed of astrocytes express at least GDNF, NRTN, GFRα1, and GFRα2 (Rémy et al., 2001). Moreover, GFRα1 and GDNF are released by the reactive astrocytes (Bresjanac and Antauer, 2000; Marco et al., 2002).

Microglial cells express GFRα1 and RET (Honda et al., 1999; Boscia et al., 2009, 2013; Rickert et al., 2014) and also synthesize and secrete GDNF (Matsushita et al., 2008). GFLs have proven effects in these cells (Rocha et al., 2012; Rickert et al., 2014). For example, Rickert et al. (2014) showed that GDNF, NRTN, ARTN and PSPN are able to reduce nitric oxide production by microglial cells and decrease mRNA levels of IL-1β, TNF-α, IL-6, and Cox-2 in these cells. Thus, this study proves that at least *in vitro* GFL members interfere with the synthesis and release of pro-inflammatory and neurotoxic molecules generated by the activated microglia (Rickert et al., 2014) implying active involvement of GFL in quenching neuroinflammation. Rocha et al. (2012) in the culture of midbrain microglia stimulated with zymosan A, demonstrated that astrocytic GDNF is able to inhibit the activation of microglia, namely, its phagocytic activity and the production of reactive oxygen species, since both parameters were greatly reduced in cells incubated with an astrocyte conditioned medium. To evaluate the nature of the soluble mediators astrocyte conditioned media was treated with specific antibodies against GDNF, CDNF, and BDNF in order to specifically sequester each of the above-mentioned NTFs and thus revert the changes induced by their presence (Rocha et al., 2012).

Chang et al. (2006) showed that GDNF has a positive regulatory effect on functional activity of primary rat microglia increasing the enzymatic activity of superoxide dismutase (SOD), expression of surface antigen intercellular adhesion molecule-1 (ICAM-1), the production of the integrin alpha5 subunit, and the phagocytic capability (Chang et al., 2006). GDNF was also able to support the survival of primary microglia *in vitro* (Salimi et al., 2003).

Matsushita et al. (2008) investigated the ability of microglia to produce and secrete GDNF *in vitro*. Secretion, but not synthesis of GDNF, was strongly suppressed in LPS-stimulated microglia. Because the stimulation of microglia with LPS led to the accumulation of GDNF in the cells as well as their morphological activation, it is plausible to hypothesize that the deposition of GDNF in the cells may be necessary for the process of microglial activation. The suppression of GDNF secretion was mediated by protein kinase C alpha (PKCα) and mitogen-activated protein kinases (MAPK) signaling cascades (Matsushita et al., 2008).

Apart from the direct effects on microglial and astroglial cells, GDNF modulates the relationships between microglia and astrocytes and astrocytes and neurons. Microglia-derived GDNF protected astrocytes against *in vitro* ischemia-induced damage (deprivation of glucose, oxygen, and serum) (Lee et al., 2004; Lu et al., 2005). GDNF inhibited the apoptosis of the ischemic astrocytes via upregulation of expression of extracellular signal-regulated kinase (ERK1/2) and nuclear factor-kappa B (NF-kB) pathways in a caspase 3-independent manner (Chu et al., 2008; Zlotnik and Spittau, 2014).

GDNF released from astroglia protected neurons from inflammation-induced neurotoxic damage, showing that at least in this condition the astrogliosis is principally neuroprotective (Chen et al., 2015).

In deafferentation pain model the bone marrow mesenchymal stem cells (BMSC) inhibited neuroinflammation by transforming microglial destructive M1 phenotype into regenerative M2 phenotype, thus alleviating pain. This process likely occurred via GDNF-induced suppression of NF-κB and activation of PI3K/AKT signaling pathways (Zhong et al., 2020).

In addition to neurons and glia GDNF can also influence non-nervous system cells. Endothelial cells may also express GFRα1 and GFRα2 as it was shown for the cells composing blood-retinal and blood brain barriers (Igarashi et al., 1999, 2000). GDNF also enhanced the phagocytic activity of the macrophages via GFRα1 in a Ret-independent manner (Hashimoto et al., 2005).

A recent review by Duarte Azevedo et al. (2020) compiled information regarding GDNF and its production in health and disease in the central nervous system. The authors conclude that neuroinflammation induces GDNF expression in activated astrocytes and microglia, infiltrating macrophages, nestin-positive reactive astrocytes, and neurons/glia (NG2)-positive microglia-like cells (Duarte Azevedo et al., 2020). In addition, RET and GFRα1 expression in glia is upregulated thus, GDNF can produce direct effects in these cells (Duarte Azevedo et al., 2020).

One important direction of pain research is detecting sex differences, deciphering the mechanisms of these differences in the development and maintenance of neuropathic pain and the analysis of sex difference in response to analgesics. Routine animal studies have generally been conducted in males mainly to avoid the influence from cyclic sex hormone fluctuations occurring in females and thus to reduce animal usage and overcome funding limitations. However, there are numerous reports in literature indicating that neuropathic pain is more common in women. Moreover, women tend to be less responsive to analgesics. While the scope of this review does not allow us to go deep into this topic, a nice summary of the available data and hypotheses explaining sex differences in pain perception can be found in the reviews and editorials authored and co-authored by J. Mogil (Craft et al., 2004; Mogil and Bailey, 2010; Mogil, 2016, 2020; Rosen et al., 2017).

Given to the fact of sex-bias in the recent years the number of animal studies deciphering sex differences in pain has substantially increased (Bartley and Fillingim, 2013). Despite of the efforts final conclusions on the mechanisms of sex-specific differences in pain are yet to be reached. In experimental animals both sexes show identical morphological reactivity of microglia (i.e., microgliosis) after nerve injury. However, microglia inhibitors are anti-nociceptive only in male rodents (Sorge et al., 2015). The sex-specific differences in GFLs and GFL receptors expression are not clearly described. Lopes et al. (2017) in a recent study conducted using RNA-seq methodology failed to detect clear sex-bias in the expression of neuronal or glial markers in somatosensory system, but reported a difference in expression of peripheral immune cell markers (Lopes et al., 2017). Further studies of sex difference in pain are essential to understand the mechanisms and develop efficient analgesics.

## Targeting Gfl Receptors With Small Molecules or Proteins Downregulate Glial Marker Expression in Surgery and Diabetes-Induced Neuropathy

In line with the literature described above, our preliminary experimental results also support the notion that GFLs or compounds targeting GDNF receptor RET (Bespalov et al., 2016; Sidorova et al., 2017; Viisanen et al., 2020) can reduce the expression of satellite glia marker in sensory ganglia of animals with surgery or diabetes-induced neuropathy. We developed RET agonists (compounds BT18 and BT44) and showed that they, similarly to GDNF and ARTN, activate RET and downstream signaling in immortalized cells. In surgery- and diabetes-induced models of neuropathic pain in rats both of these compounds alleviated mechanical hypersensitivity. They also protected and restored expression of sensory neuron markers in DRGs *in vivo* (Bespalov et al., 2016; Sidorova et al., 2017; Viisanen et al., 2020). We evaluated the expression of glial marker Ionized Calcium-Binding Adapter Molecule 1 (IBA1) in DRGs of rats with neuropathies after the treatment with ARTN or RET agonists (experimental design and sample collection procedures, as well as the effects of compound on pain threshold and on neurons are described in details elsewhere (Sidorova et al., 2017; Viisanen et al., 2020) using immunohistochemistry method described in details in Jokinen et al. (2018). Briefly, 5 micrometers sections of DRGs were probed with antibodies against IBA1 (1:1000, Cat. No. 019-19741, Wako, Richmond, VA, USA). For visualization of the bound antibodies we used a VECTASTAIN ABC HRP Kit according to the manufacturer's instructions. Sections were imaged with help of 3DHISTECH Scanner (3DHISTECH Ltd, Budapest, Hungary). Image analysis (the number of IBA1-positive cells and the area covered by IBA1-positive staining) was performed with Matlab R2014b software (Mathworks, Natick, MA, USA) using a custom script (Penttinen et al., 2016; Jokinen et al., 2018). The data collected from two non-consecutive sections per animal were averaged and used for statistical analysis by ANOVA with Dunnett's *post-hoc* test. Each group included 3–5 animals.

**Figure 2:**
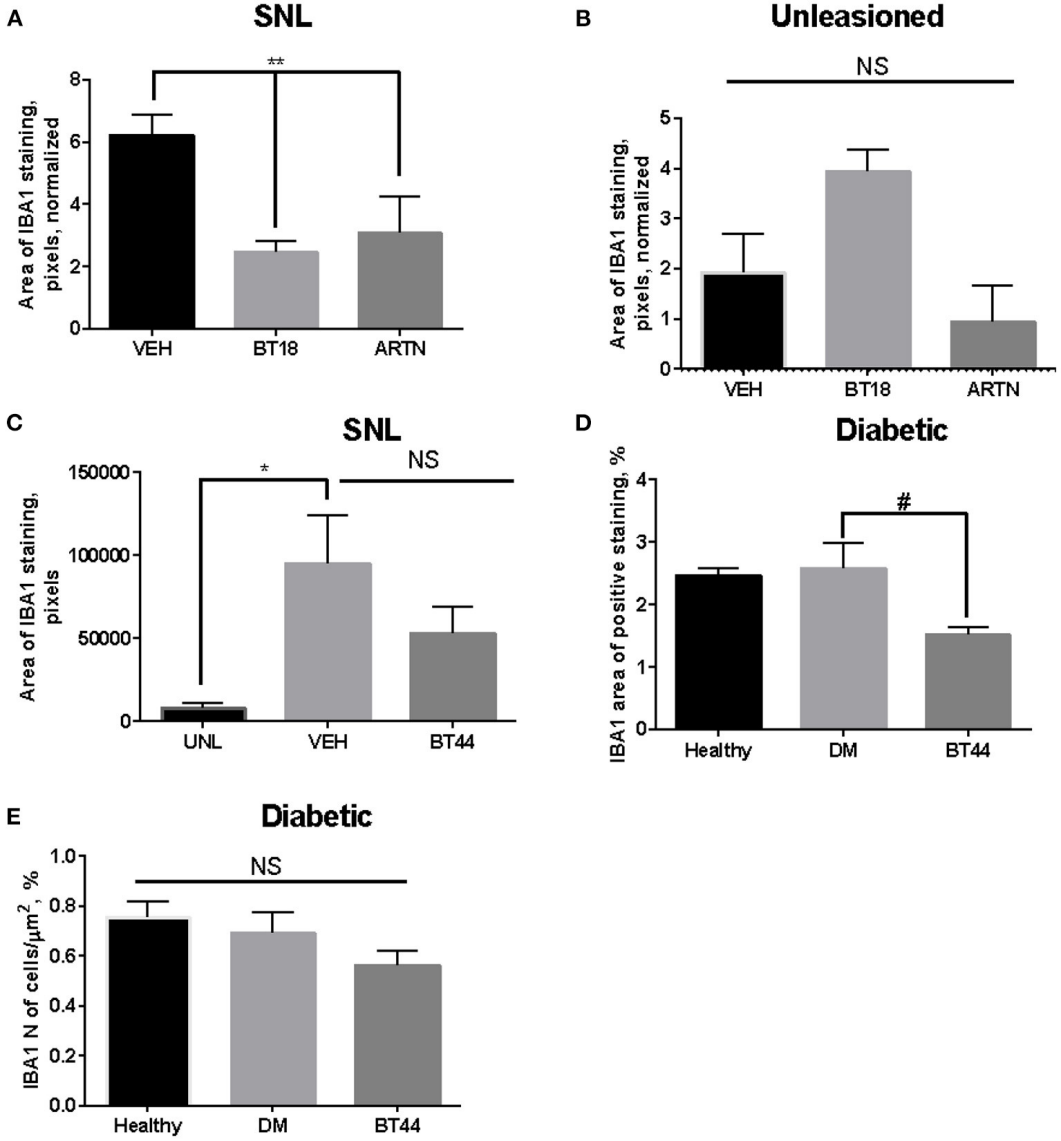
Expression of IBA-1 in dorsal root ganglia (DRG) of animals with surgery (spinal nerve ligation, **A–C**) or diabetes-induced neuropathy **(D,E)**. **(A)** Expression of IBA1 (area) in DRGs which spinal nerve was ligated upon the treatment with vehicle (VEH), BT18 (the first generation RET agonist, subcutaneously at the dose 25 mg/kg every other day for 12 days, started 1 h after the lesion), and ARTN (at the dose 0, 5 mg/kg every other day for 12 days, started 1 h after the lesion), *n* = 3 per group. **(B)** Expression of IBA1 in DRGs on the unligated side of the body (healthy), the treatments and doses are the same as in **(A)**, *n* = 3 per group. **(C)** Expression of IBA1 (area) in DRGs which spinal nerve was ligated upon the treatment with vehicle (VEH) or BT44 (the second generation RET agonist, subcutaneously at the dose 12.5 mg/kg every other day for 12 days, started 2 days after the lesion) or on unleasoned side (UNL), *n* = 3–4 per group. **(D)** Expression of IBA1 (area) in DRGs of healthy animals (healthy) or animals with diabetes-induced neuropathy treated subcutaneously with vehicle (DM) or BT44 (at the dose 5 mg/kg every other day for 3 weeks started on the day of lesion), *n* = 4–5 per group. **(E)** As in **(D)**, but the number of cells is calculated, *n* = 5 per group, *p < 0.05, **p < 0.01 by ANOVA and *post-hoc* test, #*p* < 0.05 by *post-hoc* test (ANOVA *p* = 0.059).

In our pilot experiments we consistently saw a decrease in the area and/or number of IBA1-positive cells in DRGs of animals with experimental neuropathies treated with ARTN or RET agonists compared to the values seen in DRGs of animals treated with vehicle (Figure 2). In some of these experiments this reduction was statistically significant, while in others we saw only a trend to a decrease. In ipsilateral DRGs of rats with spinal nerve ligation-induced neuropathy the area of IBA1-positive staining was ~3 times higher compared to that in contralateral DRGs. The treatment of rats with spinal nerve ligation (SNL) with either BT18 or ARTN every other day except weekends for a 12-days period starting 1 h after the surgery (Bespalov et al., 2016) resulted in a statistically significant decrease in the area covered by IBA1-positive signal in ipsilateral DRGs (Figure 2A). At the same time in contralateral DRGs statistically significant changes in the expression of studied marker were not detected (Figure 2B). In another experiment we also saw a statistically significant increase in IBA1 expression in ipsilateral DRGs after spinal nerve ligation (Viisanen et al., 2020). The treatment with a RET agonist, compound BT44, which started 2 days after the surgery when neuropathy was already established, partially normalized this change, although statistically significant decrease was not seen (Figure 2D). In rats with diabetic neuropathy induced by streptozotocin (STZ) administration (Viisanen et al., 2020) BT44 treatment resulted in the decrease in the area (Figure 2D), but did not influence in the number of IBA1-positive cells (Figure 2E). This result is fully in line with recently published data of Ciglieri et al. (2020). These authors observed cytoarchitectonic changes related to glia-neuronal interactions in DRGs upon the treatment with STZ, while the overall number of SGC remained largely unaffected. In particular, STZ differentially affected the expression of SGC marker surrounding different subpopulations of DRG neurons and disturbed glia-neuron and neuron-neuron contacts in this tissue (Ciglieri et al., 2020).

To summarize, our data taken together are in line with published observations on the ability of GFLs to downregulate activation of glial cells and mitigate neuroinflammation described above. While clinical translation of GFL proteins is complicated, small molecules targeting their receptors (Sidorova et al., 2017; Jmaeff et al., 2020a,b; Viisanen et al., 2020) may be a better alternative for use in humans to treat neuropathic pain. Previously we showed that RET agonists are neuroprotective/neurorestorative in animal models of neuropathic pain (Sidorova et al., 2017; Viisanen et al., 2020). Here, we provide evidence that these small molecules can also downregulate glial cells activation being thus dual action agents.

## Summary and Conclusions

GFLs attracted a lot of attention of scientific community as potential disease-modifying treatments against neurodegeneration due to their well-known neuroprotective effects in various neuronal populations. In the present paper we reviewed published and provided our own data showing that GFLs can also modulate neuroinflammation which accompanies and exaggerates neurodegeneration. Based on the available data regarding the expression of GFL receptors in various cell types it is reasonable to conclude that these proteins may directly influencing immune, endothelial and glial cells, and also regulate their interactions. The ability of GFLs to influence both neuronal and glial cells further increases their attractiveness as targets for the development of novel treatments against neurodegeneration. While the use of GFL proteins in humans is complicated, small molecules that target GFL receptors may serve as better alternatives for clinical translation. We developed compounds that target the main GFL receptor RET and possess both neuroprotective properties and an ability to suppress glial activation in sensory system. Further optimization of these compounds can result in a disease-modifying drug normalizing the function of all cell types involved in the process of pain chronization, which will revolutionize neuropathic pain management.

## Author Contributions

AK and YS collected, analyzed and interpreted the data, wrote the draft of the manuscript, and approved its final version. YS conceived the idea of the manuscript. All authors contributed to the article and approved the submitted version.

## Conflict of Interest

YS is a minor shareholder of GeneCode Ltd, a company owning a patent for BT compounds. The remaining author declares that the research was conducted in the absence of any commercial or financial relationships that could be construed as a potential conflict of interest.
